# Structural brain alterations associated with brain age may link to social dysfunction in male adults with autism spectrum disorder

**DOI:** 10.3389/fnins.2026.1795744

**Published:** 2026-07-28

**Authors:** Gang Xiao, Xiaoshi Li, Yue Qin, Wanting Zhao, Xin Li, Yifan Qian, Juan Tian, Xueting Chen, Wei Li, Lei Wang

**Affiliations:** 1 Department of Radiology, Xi’an Daxing Hospital Affiliated to Yan’an University, Xi’an, China; 2 Department of Radiology, The First Affiliated Hospital of Xi’an Jiaotong University, Xi’an, China; 3 Shaanxi University of Chinese Medicine, Xianyang, China; 4 Department of Radiology, Tangdu Hospital, The Fourth Military Medical University, Xi’an, China

**Keywords:** autism spectrum disorder, brain age gap, combined batch effect correction, fractal dimension, gray matter volume

## Abstract

**Background:**

While atypical brain development in autism spectrum disorder (ASD) has been extensively characterized during childhood and adolescence, it remains unclear how these neurodevelopmental deviations persist into adulthood and affect brain aging. Existing studies relying on single morphometric measures have yielded inconsistent findings, underscoring the need for integrative, multiscale neuroimaging approaches.

**Materials and methods:**

Using data from the Autism Brain Imaging Data Exchange I (ABIDE-I) dataset, we investigated brain structural alterations in 90 adult males with ASD and 132 age-matched typically developing (TD) controls. All participants were right-handed and aged 18–55 years. Voxel-based morphometry (VBM) was employed to assess gray matter volume (GMV), and surface-based morphometry (SBM) was used to quantify cortical fractal dimension (FD). Global brain aging was evaluated using MRI-derived brain age estimation, from which the brain age gap (BAG) was calculated. Site-related effects were harmonized using the ComBat method. Group comparisons were performed for GMV, FD, and BAG using multiple linear regression, with age, full-scale IQ, and total intracranial volume included as covariates. Associations between neuroimaging metrics and Autism Diagnostic Observation Schedule (ADOS) scores were further examined.

**Results:**

Cross-sectional comparisons demonstrated that adults with ASD exhibited higher estimated BAG values relative to TD controls (*F* = 6.838, *p* = 0.01, partial η^2^ = 0.031). ComBat-harmonized morphometric analyses revealed exploratory localized GMV and FD differences, including increased GMV and FD in the right precuneus and increased FD in the lingual gyrus and lateral orbitofrontal cortex. GMV in the right precuneus showed an exploratory positive correlation with ADOS social-domain scores (*r* = 0.214, *q* = 0.044).

**Conclusion:**

Adults with ASD exhibited higher estimated BAG relative to TD controls in this cross-sectional sample. An exploratory association between right precuneus GMV and ADOS social-domain scores suggests a possible link between localized structural variation and social symptom severity, although this finding requires replication in longitudinal and clinically richer datasets given their sensitivity to the harmonization strategy.

## 1 Introduction

Autism spectrum disorder (ASD) is a heterogeneous neurodevelopmental condition characterized by impairments in social communication, repetitive and stereotyped behaviors, restricted interests, and sensory abnormalities. ASD is known to have inherent patterns of atypical brain development (Chi et al., 2025; Lange et al., 2015). Over the past decade, neuroimaging studies have identified relatively consistent abnormalities in brain structure and development in children and adolescents with ASD (Pagnozzi et al., 2018; Wang et al., 2022; You et al., 2024). In contrast, structural neuroimaging investigations focusing on adults with ASD remain comparatively limited. As noted in comprehensive reviews and diagnostic guidelines, adult ASD research faces persistent bottlenecks in recruitment and diagnostic characterization due to complex co-occurring psychiatric conditions and varied intervention backgrounds (Lai et al., 2014; Lever and Geurts, 2016; Lord et al., 2020). Given the lifelong nature of ASD, examining brain structure in adulthood is critical for understanding whether early neurodevelopmental alterations translate into atypical brain aging or altered maturation trajectories later in life (Alpert, 2021).

Social impairment represents a core clinical feature of ASD and is central to its diagnostic criteria (Tafolla et al., 2025). Deficits in social communication and interaction in ASD are associated with to underlying neurodevelopmental alterations and are strongly associated with long-term functional outcomes, representing a major determinant of adaptive functioning across the lifespan (Pelphrey et al., 2011; Tamrouti-Makkink et al., 2004). Examining the relationship between neuroimaging-derived metrics and social-domain measures may help characterize potential brain–behavior associations in adults with ASD.

These social impairments have been associated with large-scale structural brain differences, and several studies have reported widespread neuroanatomical in large-scale brain structural alterations, several studies have reported widespread neuroanatomical differences in adults with ASD, including regional increases in gray matter volume (GMV) in temporal and frontal cortices, as well as widespread white matter abnormalities associated with core autistic traits and behavior (Ecker, 2012; Libero et al., 2015; Sato et al., 2017). Conversely, other voxel-based morphometry (VBM) studies have identified reduced gray matter in regions implicated in social cognition, such as the fusiform gyrus, amygdala, and prefrontal areas in adult ASD (Sato et al., 2017). However, meta-analytic evidence indicates that these findings are heterogeneous. Both increases and decreases in GMV have been reported across temporal, parietal, and frontal regions, as well as in the anterior cingulate cortex and cerebellum, underscoring the inconsistency of macrostructural results in adult ASD (Guo et al., 2024; Yang et al., 2016). For example, region-specific volumetric alterations have been documented in the posterior hippocampus, cuneus, and various prefrontal subregions in high-functioning adult ASD cohorts, yet these patterns are not consistently replicated across studies (Eilam-Stock et al., 2016). A recent large-sample neuroimaging study further reported heterogeneous and region-specific structural patterns across adulthood in individuals with ASD (Pretzsch et al., 2024). Collectively, these findings suggest that investigations of brain aging in ASD should incorporate both whole-brain and region-specific assessments.

Brain age estimation has emerged as a powerful framework for characterizing global brain aging. This approach predicts an individual’s biological brain age from neuroimaging features, and resulting brain age gap (BAG)–defined as the difference between predicted brain age and chronological age–quantifies deviations from normative age-related developmental and aging trajectories (Cole et al., 2017). Brain aging models has been widely applied in neuropsychiatric disorders, including schizophrenia, bipolar disorder, and attention-deficit/hyperactivity disorder (ADHD), where higher estimated BAG has been consistently reported (Chang et al., 2025; Hajek et al., 2019; Tønnesen et al., 2020).

At the regional level, VBM and surface-based morphometry (SBM) are among the most common approaches for assessing structural alterations. VBM enables voxel-wise quantification of gray matter morphology across individuals in a standardized anatomical space (Pergher et al., 2019). Although VBM has been extensively applied in ASD research, findings in adult cohorts remains inconsistent. For instance, one study reported no structural differences between adults with ASD and neurotypical controls (Koolschijn and Geurts, 2016), whereas another identified reduced prefrontal GMV in ASD using a similar methodology (Koolschijn and Geurts, 2016).

Surface-based morphometry reconstructs the cortical surface and allows vertex-wise analysis of cortical morphological features. Fractal dimension (FD), a surface-based metric derived from cortical geometry, quantifies the complexity of cortical folding patterns (Beltrán et al., 2024). SBM studies in ASD have reported heterogeneous cortical abnormalities, including altered FD in social cognition (Libero et al., 2014), as well as age-dependent variations in cortical complexity (Shen et al., 2024). Such discrepancies likely reflect differences in age range, sample composition, and analytical strategies, highlighting the need for integrative, multi-metric approaches.

Importantly, it remains unclear whether global deviations in brain aging and regional gray matter abnormalities in adult ASD represent convergent manifestations of a shared neurobiological process or dissociable structural mechanisms operating at different spatial scales. Addressing this question requires a framework that integrates global aging indices with regional morphometric measures. Such an approach may help to identify neuroanatomical patterns associated with clinically relevant heterogeneity and aging-related vulnerability in adulthood, thereby providing a foundation for future longitudinal and translational studies.

Accordingly, the present study combines MRI-derived brain age estimation with VBM-based GMV and SBM-based FD analyses to characterize both global and regional structural alterations in adults with ASD. Given that social impairment is a hallmark of adult ASD and is consistently associated with alterations in “social brain” circuits–including the precuneus and temporal regions–we hypothesized that multiscale structural metrics would primarily correlate with the ADOS social domain. Specifically, we aim to: (1) examine whether adults with ASD exhibit altered brain aging relative to typically developing individuals; (2) identify regional gray matter alterations associated with ASD in adulthood; and (3) investigate the relationships between structural imaging markers and ASD symptom dimensions, particularly social dysfunction.

## 2 Materials and methods

### 2.1 Participants

Neuroimaging and phenotypic data were obtained from the Autism Brain Imaging Data Exchange I (ABIDE-I) database (Di Martino et al., 2014). Inclusion criteria were as follows: (1) male sex; (2) age 18–55 years; (3) right-handedness; (4) availability of high-quality T1-weighted structural MRI data; and (5) complete demographic and diagnostic information. Participants with excessive head motion, gross anatomical abnormalities, or incomplete imaging data were excluded. Data from 9 ABIDE-I sites were included in the present study. The site-wise sample sizes, exclusion procedures, and other detailed information were provided in Supplementary Table 1.

**FIGURE 1:**
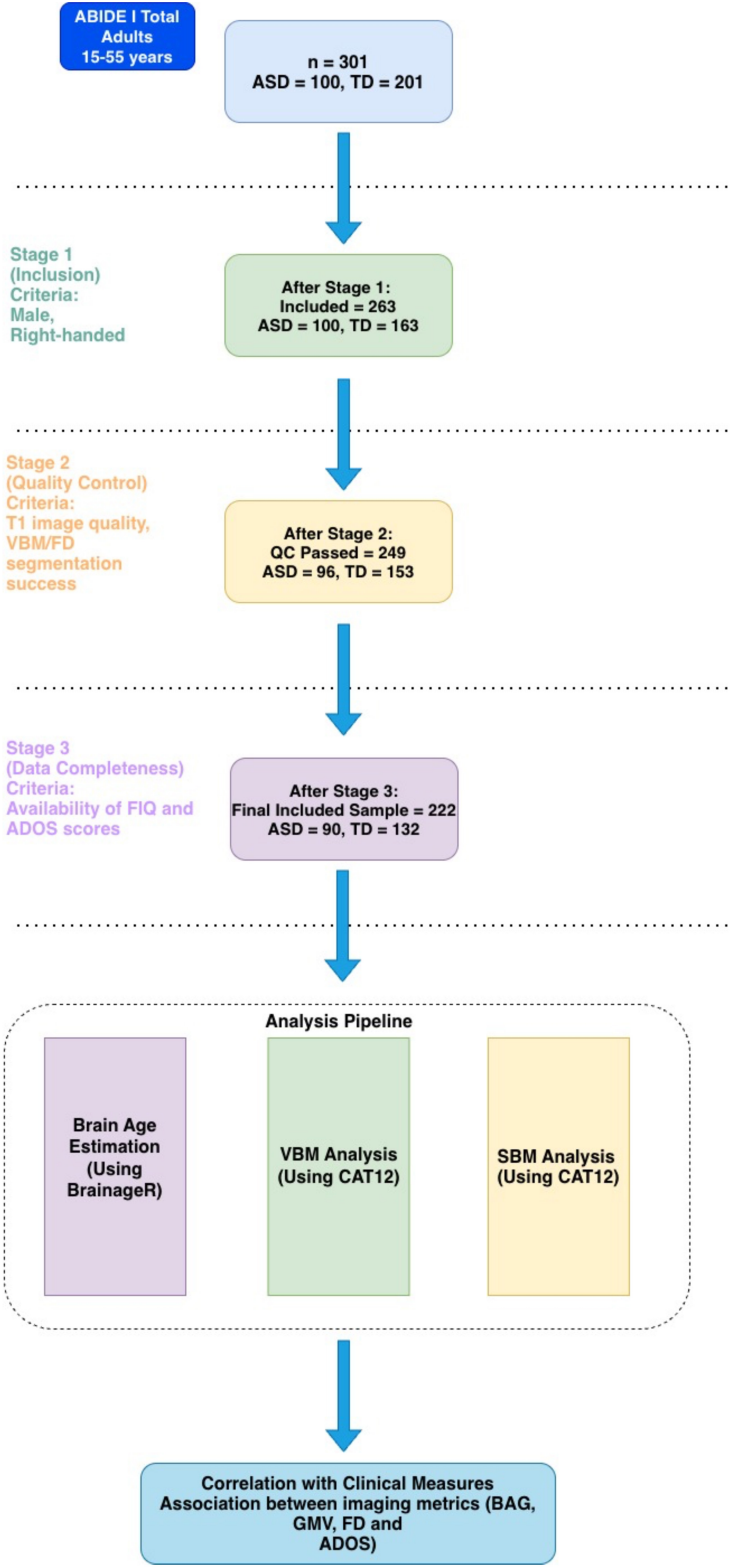
Flowchart of participant selection, quality control procedures, and neuroimaging analysis pipeline in the present study. Adult participants from the ABIDE I database underwent staged screening based on demographic criteria, image quality control, and data completeness. The final sample was included for brain age estimation using brainageR and structural analyses using CAT12, including VBM and SBM analyses. Associations between imaging-derived metrics and clinical symptom severity were further evaluated using ADOS measures.

The final sample comprised 90 adult males with ASD and 132 age-matched typically developing (TD) controls. All ASD diagnoses were confirmed using the Autism Diagnostic Observation Schedule (ADOS), according to site-specific protocols. Dataset preparation and the subsequent workflow are illustrated in Figure 1.

### 2.2 Structural MRI preprocessing

All structural MRI data were processed using the Computational Anatomy Toolbox (CAT12, version 12.8.2) (Gaser et al., 2024) implemented in Statistical Parametric Mapping (SPM12, v7771)^1^ running on MATLAB R2023a. Both VBM and SBM analyses were conducted following standard CAT12 pipelines and methodological recommendations (Gaser et al., 2024). The quality control threshold was strictly set to exclude any image with a CAT12 weighted quality score below 75% (equivalent to a QC grade below “B−”).

#### 2.2.1 VBM analysis

T1-weighted images were bias-field corrected, tissue-segmented into gray matter (GM), white matter, and cerebrospinal fluid and spatially normalized to the Montreal Neurological Institute (MNI) space. GMV maps were modulated to preserve volume information and smoothed with an 8-mm full-width at half-maximum (FWHM) Gaussian kernel consistent with standard VBM practice and CAT12 recommendations (Dahnke et al., 2013; Gaser et al., 2024). All structural images underwent both visual inspection and CAT12’s automated quality assessment.

Total intracranial volume (TIV) was estimated for each participant and included as a covariate in subsequent statistical analyses to control for individual differences in head size.

#### 2.2.2 SBM analysis and FD estimation

Cortical surface reconstruction was performed in CAT12 through automated skull stripping, topology correction, and extraction of the white and pial surfaces, followed by spherical inflation. Individual cortical surfaces were mapped onto a sphere and registered to the CAT12 standard symmetric surface template using sulcal depth–based spherical registration, ensuring accurate vertex-wise correspondence across participants. FD estimation was performed using the default CAT12 pipeline with default spherical harmonics parameters, Vertex-wise FD maps were spatially smoothed using a 15-mm FWHM Gaussian kernel and projected onto a common surface space for group-level analyses, Surface-based FD maps were smoothed with a 15-mm FWHM Gaussian kernel. This kernel size was chosen to optimize the balance between capturing local morphological complexity and satisfying the topological requirements for vertex-wise group comparisons on the standardized surface (Yan et al., 2024).

### 2.3 BAG analysis

The brain age model was implemented via R environment 4.3.2 and the brainageR framework (version 2.1)^2^, which was pretrained on a large-scale reference dataset of healthy individuals (*n* = 3,377; age range: 18–92 years) using Gaussian Processes Regression (Cole et al., 2017). The training sample included a diverse range of scanner types and protocols from multiple open-access repositories, ensuring the model’s generalizability. This age-matched training range is appropriate for our adult ASD cohort (18–55 years). This approach has been successfully applied to detect higher estimated brain aging and structural deviations in various psychiatric conditions, including schizophrenia, bipolar disorder (Tønnesen et al., 2020), posttraumatic stress disorder (Clausen et al., 2022), major depressive disorder (Wagenmakers et al., 2023) and childhood psychiatric trauma (Hendrikse et al., 2025). Its scope further extends to systemic health, capturing neuro aging patterns in metabolic syndrome (Qin et al., 2026). Such widespread application highlights the framework’s robustness and adaptability as a reliable indicator of brain health in diverse research contexts.

Briefly, the preprocessed GM maps were smoothed with a 4-mm FWHM Gaussian kernel to enhance the signal-to-noise ratio and reduce inter-individual anatomical variability. The smoothed voxel-wise GMV were then used as input features for a trained regression model. Finally, the trained model was then applied to the ASD and TD participants to generate individual predicted brain ages.

The BAG was computed for each participant as:


$$\text{B}\,A\,G_{i}=\text{B}\,r\,a\,i\,n\,a\,g\,e_{i}-C\,h\,r\,o\,n\,o\,l\,o\,g\,i\,c\,a\,l\,a\,g\,e_{i}$$

B*rainage*_*i*_ where represents the predicted brain age and *Chronologicalage*_*i*_ is the actual age of participants. A positive BAG indicates higher estimated brain aging relative to chronological age, whereas a negative BAG suggest delayed or preserved brain aging (Cole et al., 2018).

### 2.4 Harmonization

To mitigate site-related variability associated with multi-center data acquisition, harmonization procedures were applied to all imaging-derived measures prior to statistical analyses. Specifically, GMV and FD maps were harmonized using the DPABI (v 9.0) harmonization module (Wang et al., 2024), whereas BAG values were harmonized using the ComBat (Combined Batch Effect Correction) method (Fortin et al., 2018) via the GitHub ComBat package^3^. ComBat implements an empirical Bayes framework that adjusts for systematic differences in feature distributions by modeling and correcting location (mean) and scale (variance) effects associated with each site while preserving biological variation of interest (e.g., diagnosis, age) (Ingalhalikar et al., 2021). For each feature set, site labels were specified as batch variables, age and diagnosis were included as biological covariates to preserve variation associated with these factors during harmonization (Chandio et al., 2024). Harmonized data were subsequently used for statistical analyses to reduce site-related variability and improve comparability across acquisition sites while attempting to preserve biological variation of interest (Chen et al., 2022; Ma et al., 2024).

### 2.5 Statistical analysis

Demographic and clinical characteristics were analyzed using SPSS (IBM Corp., Armonk, NY, USA, version 29.0), continuous variables were summarized as mean ± standard deviation (SD) and compared between groups via two-sample *t*-tests, while categorical variables were tested using χ^2^ tests.

Statistical analyses were performed on harmonized data after removal of site effects. Group differences in BAG were assessed using multiple linear regression models, controlling for age, full-scale IQ (FIQ), and TIV.

Gray matter volume and FD were computed within the CAT12 framework and analyzed using general linear models controlling for age, FIQ and TIV. GMV results were corrected using Gaussian Random Field (GRF) theory at the cluster level (voxel-level *p* < 0.001, cluster-level *p* < 0.05, two-tailed), whereas FD results were corrected using false discovery rate (FDR) at the whole-brain level (q < 0.05, two-tailed).

Correlation analyses between GMV, FD, and ADOS scores were performed using partial Pearson correlation, controlling for age, FIQ, and TIV, with multiple comparisons corrected using the FDR (q < 0.05, two-tailed).

To examine group differences in BAG, a multiple linear regression model was constructed with BAG as the dependent variable and Group (ASD vs. TD) as the primary predictor. Chronological age, FIQ, and TIV were included as covariates to isolate the specific effect of diagnosis.

### 2.6 Sensitivity analyses

To evaluate the robustness of the primary findings, a series of supplementary sensitivity and diagnostic analyses were performed.

First, brain age model performance was evaluated separately in the ASD and TD groups using the mean absolute error (MAE), mean prediction error (MPE), and the correlation between predicted and chronological age. Correlations between BAG and chronological age were additionally examined in the overall sample and within each diagnostic group.

Second, a sensitivity analysis excluding FIQ as a covariate was additionally performed to test the statistical dependency of the primary analytical framework, ensuring that the main effect of diagnosis on the estimated brain-age gap was not driven by the specific covariate structure or the inclusion of general cognitive metrics.

Third, site-stratified evaluations of brain age model performance were conducted across major imaging sites, and parallel analyses without ComBat harmonization were additionally performed.

Fourth, to ensure that the primary group-level inferences were not artifacts artificially introduced or inflated by the ComBat framework, a parallel sensitivity analysis was performed on the raw, unharmonized brain metrics using a standard ANCOVA framework with site treated as a nuisance covariate.

Fifth, to rigorously stress-test the empirical Bayes model’s stability against site-imbalance anomalies, an iterative leave-site-out sensitivity analysis was conducted by fully excluding data from individual sites characterized by single-group architectures or highly asymmetric diagnostic cell distributions (specifically, the LEUVEN_1 and MAX_MUN cohorts). The core regression models were then re-estimated to confirm the statistical persistence of the diagnostic group effects.

Sixth, a series of diagnostic analyses were performed to evaluate residual normality, homoscedasticity, and the presence of influential observations.

Finally, the primary statistical analyses were repeated after excluding extreme observations exceeding ±2.5 standard deviations from the group mean to evaluate the robustness of the results to potential outliers.

## 3 Results

### 3.1 Demographics and clinical information

A final sample of 222 participants (90 ASD, 132 TD) was included in all subsequent VBM, FD, and BAG analyses. The TD group showed significantly higher FIQ scores compared with the ASD group (Table 1), given the significant group difference in FIQ (*t* = −5.25, *p* < 0.001), FIQ was consistently included as a nuisance covariate in all subsequent neuroimaging group comparisons.

**TABLE 1:** Demographic variables (*n* = 222, ASD = 90, TD = 132).

Variable	ASD (*n* = 90)	TD (*n* = 132)	*t*	*p*
Age (years)	26.46 ± 8.16	25.91 ± 6.20	0.57	0.55
Sex (male)	90	132	–	–
Full-scale IQ score	109.61 ± 8.87	112.52 ± 10.66	−5.25	<0.01*
ADOS total score	12.21 ± 3.20	–	–	–
ADOS social score	8.05 ± 2.20	–	–	–
ADOS communication score	4.50 ± 1.20	–	–	–
ADOS stereotyped behavior score	1.05 ± 0.52	–	–	–

*p < 0.05.

### 3.2 VBM analysis

**FIGURE 2:**
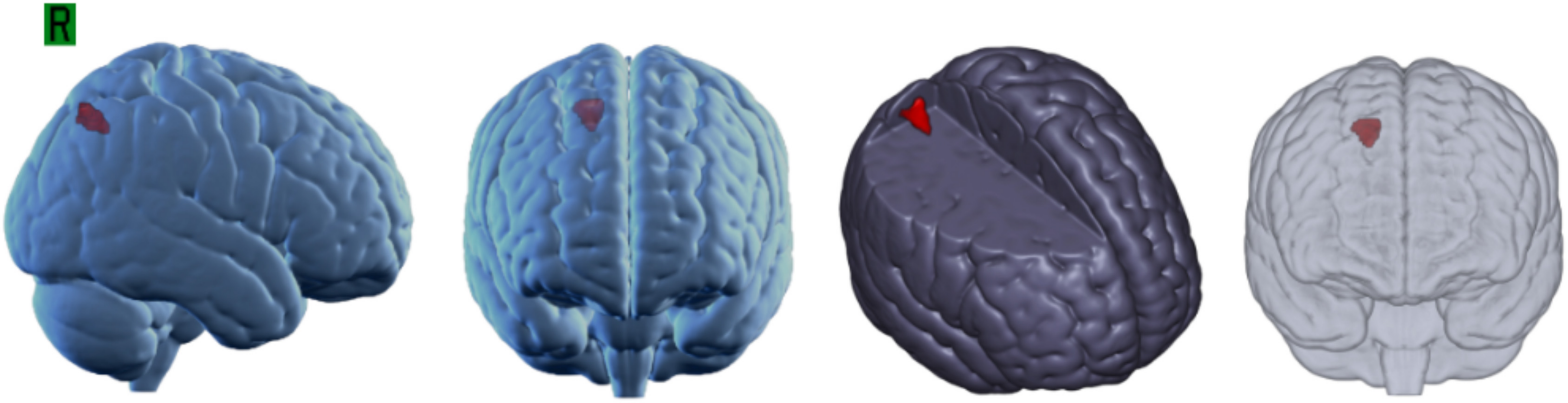
Adults with ASD showed increased GMV in the right precuneus compared with TD (voxel *p* < 0.001, cluster *p* < 0.05, *k* = 229).

Voxel-wise analyses revealed a focal increase of GMV in the right precuneus in adults with ASD relative to TD controls (Figure 2), with no other regions showing significant group differences after correction for multiple comparisons (GRF-corrected, voxel *p* < 0.001, cluster *p* < 0.05, *k* = 229).

Mean GMV within the significant cluster was higher in the ASD group compared with TD controls (ASD: 0.421 ± 0.106; TD: 0.359 ± 0.009), with a significant group difference (*p* < 0.001, *t* = 4.741, Cohen’s *d* = 0.642). Distribution inspection indicated greater variability in the ASD group.

### 3.3 SBM analysis

**FIGURE 3:**
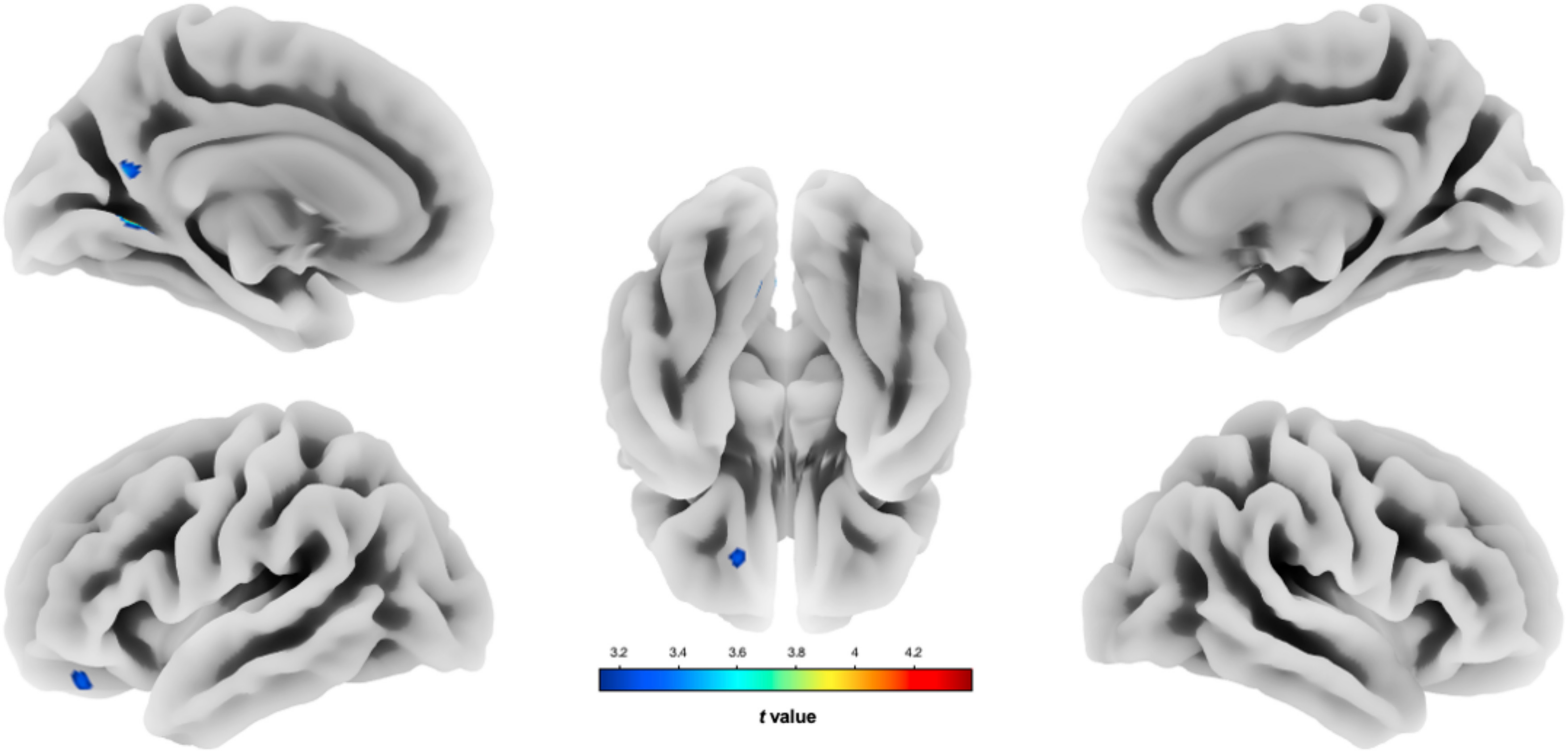
Adults with ASD showed significantly increased cortical FD in the right lingual gyrus, lateral orbitofrontal cortex, and precuneus compared with TD controls (FDR correction *p* < 0.05).

Surface-based morphometry analysis revealed significantly increased cortical FD in adults with ASD compared with TD controls. After FDR correction (cluster-level *q* < 0.05), significantly increased FD values were observed in the right lingual gyrus, right lateral orbitofrontal cortex, and right precuneus (Figure 3).

**FIGURE 4:**
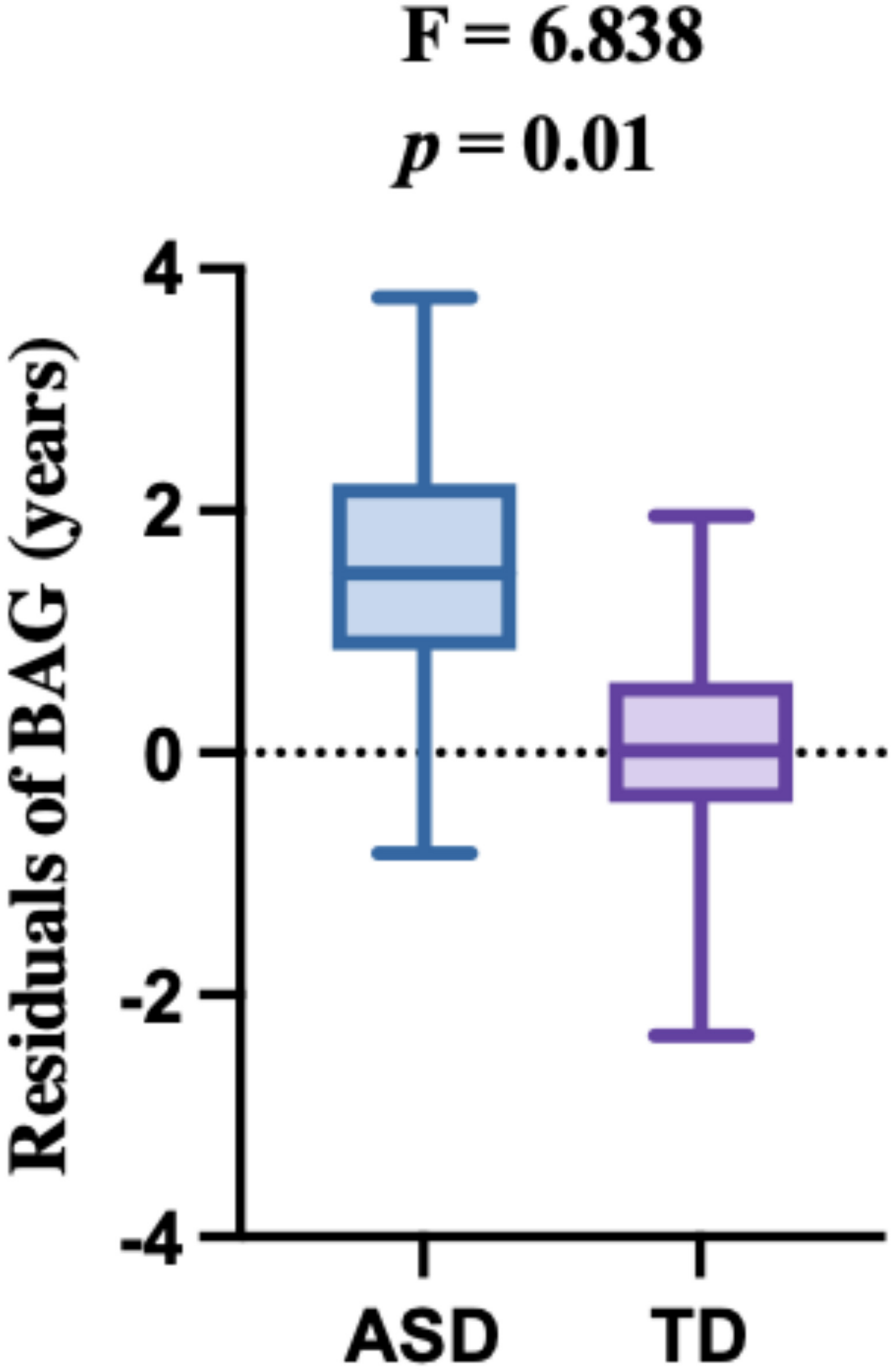
Comparison of adjusted brain age gap BAG between ASD and TD groups. The *Y*-axis represents the unstandardized residuals of BAG, derived from a linear regression model that controlled for age, FIQ, TIV, and imaging site. The mean residual for the ASD group was 1.531 ± 0.512 years, while the mean for the TD group was 0.011 ± 0.422 years. A significant main effect of diagnosis was observed (*F* = 6.838, *p* = 0.01). ASD, autism spectrum disorder; TD, typically developing.

Mean FD values within these regions were significantly higher in the ASD group compared with TD controls (right lingual gyrus: 2.499 ± 0.652 vs. 2.149 ± 0.706, *t* = 3.77, *p* < 0.001 (Figure 4); right lateral orbitofrontal cortex: 2.499 ± 0.525 vs. 2.163 ± 0.792, *t* = 3.73, *p* < 0.001; right precuneus: 2.520 ± 0.931 vs. 2.136 ± 0.820, *t* = 3.22, *p* = 0.002), indicating increased cortical folding complexity in ASD.

### 3.4 BAG analysis

Brain age gap differed significantly between groups after adjustment for site effects and covariates, with individuals with ASD showing a higher BAG than TD controls (*F* = 6.838, *p* = 0.01, partial η^2^ = 0.031). The ASD group exhibited a 1.52-year higher adjusted BAG compared to the TD group (1.53 ± 0.51 vs. 0.01 ± 0.42 years; 95% CI: 0.20–2.84). Additional details of the regression analysis are provided in the Table 2. The validity of the regression assumptions was rigorously evaluated; detailed diagnostic plots, including the Normal Q-Q plot and the residual scatterplot, are provided in Supplementary Figures 1, 2, respectively.

**TABLE 2:** Multiple linear regression model examining group differences in BAG.

Adjusted model (MLR)	B	SE	β	t	*p*	95% CI	VIF
Group (ASD vs. TD)	1.784	0.682	0.178	2.615	0.01	[0.439, 3.129]	1.057
Age	−0.044	0.046	−0.062	−0.938	0.349	[−0.135, 0.048]	1.003
Full-scale IQ (FIQ)	0.042	0.026	0.11	1.594	0.112	[−0.01, 0.094]	1.102
TIV	−0.006	0.003	−0.163	−2.388	0.018	[−0.012, −0.001]	1.062

For the “Group” variable, TD was coded as 0 and ASD was coded as 1.

The pre-trained brainageR model demonstrated high predictive performance in our sample, with a strong correlation between predicted and chronological age (*r* = 0.808, *p* < 0.001) and a Mean Absolute Error (MAE) of 3.68 years (detailed information can be found in Supplementary Table 2). These metrics are aligned with the original validation of the brainageR framework (Cole et al., 2017).

### 3.5 Correlation analysis

**FIGURE 5:**
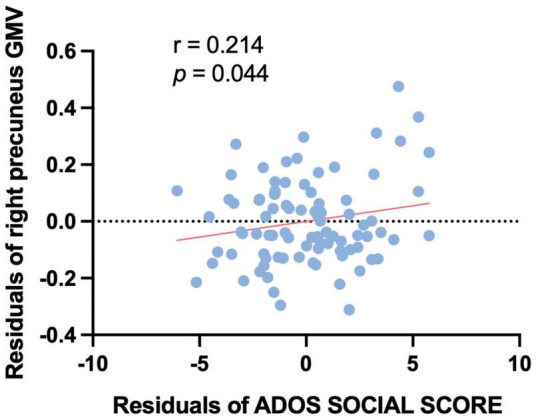
Significant positive correlation between right precuneus GMV and ADOS social score in adults with ASD (controlling for age, FIQ, and TIV. *r* = 0.214 *q* = 0.044).

Partial correlation analyses were conducted between BAG (controlling for age, FIQ, and TIV), extracted GMV and FD metrics, and ADOS scores. The GMV of the right precuneus showed a significant positive association (*r* = 0.214, *q* = 0.044) with the ADOS social score (Figure 5). In contrast, BAG showed no significant correlations with GMV or FD indices.

### 3.6 Sensitivity results

Brain age model performance was comparable across diagnostic groups, and no significant association was observed between BAG and chronological age in the overall sample.

A sensitivity analysis excluding Full-Scale Intelligence Quotient (FIQ) as a covariate was additionally performed. The group effect on BAG remained statistically significant (*F* = 5.356, *p* = 0.022, partial η^2^ = 0.024), indicating that the primary findings were not dependent on the inclusion of FIQ as a covariate.

Site-stratified evaluations of brain age model performance demonstrated generally comparable performance across the major imaging sites. In parallel, analyses based on unharmonized imaging metrics were performed to evaluate the influence of ComBat harmonization. When the primary diagnostic group comparison was repeated using the unharmonized site-adjusted model, the group difference in BAG remained statistically significant (*F* = 6.032, *p* = 0.015, partial η^2^ = 0.027). In contrast, the localized GMV and FD findings did not survive whole-brain multiple-comparison correction under the corresponding unharmonized site-adjusted analyses. These results suggest that the global BAG finding was relatively more robust across harmonization strategies, whereas the regional morphometric findings were more sensitive to the harmonization approach.

In addition, a leave-site-out sensitivity analysis excluding sites with highly unbalanced or single-group diagnostic composition (LEUVEN_1 and MAX_MUN) also yielded a significant group effect (*F* = 6.411, *p* = 0.012, partial η^2^ = 0.034), suggesting that the observed group-level difference was not driven by any single site or subset of sites, although effect estimates may vary depending on sample composition.

Model diagnostic analyses demonstrated no evidence of substantial violations of regression assumptions, including residual normality, homoscedasticity, or influential observations. These localized GMV findings did not survive whole-brain multiple-comparison correction in the corresponding unharmonized site-adjusted sensitivity analysis.

Finally, the primary analyses were repeated after excluding participants with BAG values exceeding ±2.5 standard deviations from the sample mean. The group effect remained statistically significant (*F* = 3.968, *p* = 0.048, partial η^2^ = 0.018), indicating that the results were not driven by a small number of extreme observations.

Collectively, these supplementary analyses yielded broadly consistent results across alternative statistical models and analytical strategies, providing supportive evidence for the consistency of the primary findings. Nevertheless, these analyses should be regarded as robustness checks and cannot completely exclude the possibility of residual site-related confounding. Detailed statistical results and performance metrics are provided in the Supplementary Material.

## 4 Discussion

The present study evaluates localized morphometric features and cross-sectional differences in machine-learning-derived brain-age metrics in adult males with ASD. By combining a multi-modal framework of VBM-GMV, surface-based FD mapping, and brain-age estimation, we observed cross-sectional group variations in estimated BAG values alongside exploratory ComBat-harmonized localized structural alterations in gray matter architecture. Furthermore, an exploratory association between right precuneus GMV and ADOS social-domain scores suggests a possible link between localized structural variation and social symptom severity, although this finding requires replication in longitudinal and clinically richer datasets.

### 4.1 Regional brain alterations in ASD

Our findings are consistent with previous meta-analysis, which reported increased precuneus activity or functional connectivity in ASD (Guo et al., 2024). As a core node of the default mode network (DMN), the precuneus constitutes part of the posterior parietal cortex on the medial surface of the cerebral hemisphere and is engaged in episodic memory, visuospatial processing, self-referential cognition, and higher-order processes such as metacognition and consciousness (Dadario and Sughrue, 2023). Increases in GMV are generally interpreted as reflecting atypical neurodevelopmental processes, such as altered synaptic pruning, increased dendritic arborization, or delayed cortical maturation, rather than improved functional capacity (Fehlbaum et al., 2022). Longitudinal structural MRI studies have demonstrated altered developmental trajectories in DMN-related regions in individuals with ASD, persisting into adulthood (Wang et al., 2021). Such atypical maturation may disrupt the functional integration of the precuneus within the DMN, leading to inefficient self-referential and social cognitive processing, which has been consistently implicated in ASD-related social deficits (Cong et al., 2023). However, because the localized GMV findings did not survive whole-brain multiple-comparison correction in the corresponding unharmonized sensitivity analysis, they should be interpreted as exploratory observations requiring replication in independent and better-balanced cohorts.

In addition, our research indicates that the FD of the right lingual gyrus and right lateral orbitofrontal cortex was also increased. The FD index captures variations in cortical folding patterns beyond conventional volumetric or thickness-based measures (Hahn et al., 2016) and a higher FD value indicates increased cortical morphological complexity, which reflects structural alterations in cortical development and organization rather than simple changes in tissue amount (Zueva, 2015). The lingual gyrus is primarily involved in visual processing and visual memory, whereas the lateral orbitofrontal cortex plays a central role in rewards evaluation and socio-emotional decision-making (Bzdok et al., 2016; Ingalhalikar et al., 2021). Increased FD in these regions may therefore reflect atypical maturation of perceptual and affective processing pathways that contribute to altered integration of visual and social information in ASD. These localized FD findings were observed in the ComBat-harmonized analysis but were not retained in the corresponding unharmonized sensitivity analysis, and therefore should also be considered exploratory pending independent replication.

The exploratory finding showed a potential association between the GMV of the right precuneus and ADOS social affect scores. This result is consistent with prior evidence identifying the precuneus as a central DMN hub supporting self-referential processing, mentalizing, and social cognition (Lesourd et al., 2023). Notably, a recent meta-analysis using a Bayesian reverse-inference framework reported a high probability of precuneus involvement in ASD-related gray matter alterations (Liloia et al., 2023). The anatomical location of the observed precuneus findings is broadly consistent with previous meta-analytic evidence (Liloia et al., 2023). For example, repetitive transcranial magnetic stimulation over parietal cortex regions has been linked to enhanced connectivity and reduced social deficits in ASD (Yang et al., 2023). Anatomical tractography and white-matter studies show that the precuneus forms substantial cortico-cortical connections with adjacent parietal regions, likely supporting multisensory integration, attention, and self-referential processing (Tanglay et al., 2022). Notably, this exploratory finding was limited to a single regional GMV cluster and was not observed for global BAG or SBM-derived FD metrics. Furthermore, while this exploratory association suggests that the right precuneus may represent one region showing structural variation associated with social-domain measures, cross-sectional statistical associations do not establish mechanistic causality. It is also critical to acknowledge that our clinical characterization relied primarily on ADOS scores, an instrument that provides a limited snapshot of ASD symptomatology and may not fully capture the full complexity of real-world social functioning in adults. Consequently, replication in longitudinal studies with more comprehensive behavioral assessments will be important to determine the reproducibility and clinical significance of this localized association Question 5. Together, these findings suggest that the right precuneus may represent one region showing localized structural variation associated with ADOS social-domain scores in adult ASD, although this exploratory finding requires further validation.

### 4.2 Elevated estimated BAG in ASD

This present study demonstrated a significant increase in BAG in adults with ASD compared with TD controls, indicating an overall tendency toward higher estimated BAG. This finding is consistent with previous studies reporting atypical neurodevelopmental characteristics in ASD that persist into adulthood and are associated with structural brain differences across the lifespan. Previous studies have shown that neurobiological abnormalities in ASD are not confined to childhood but can persist into adulthood and even later life, accompanied by progressive impairments in cognitive and social functioning (Ju et al., 2025). A large-scale neuroimaging study spanning a wide age range reported atypical developmental trajectories across multiple cortical and subcortical regions in individuals with ASD, highlighting systematic variations in brain structural maturation (Koolschijn and Geurts, 2016). Moreover, higher estimated BAG has been consistently observed across various neuropsychiatric disorders and is thought to be associated with long-standing neurodevelopmental alterations, reduced network integration efficiency, and cognitive decline (Koolschijn et al., 2016). Taken together with the exploratory regional morphometric findings, these results suggest that adults with ASD in this sample exhibit atypical neuroanatomical characteristics accompanied by higher estimated BAG.

While our results demonstrate a statistically significant higher estimated BAG in adult ASD, the observed effect size suggests that BAG alone cannot yet serve as a reliable individual-level diagnostic biomarker. The magnitude of the inter-group difference remained modest relative to the participants’ average chronological age and within-group variability. Indeed, the substantial overlap in BAG distributions between groups indicates that many ASD individuals maintain brain-aging profiles within the neurotypical range (Karolinska Schizophrenia Project (KaSP) et al., 2019). This overlap may be further exacerbated by the inherent heterogeneity within the adult ASD population–driven by varied intervention histories and diverse psychiatric comorbidities–which obscures a unified aging pattern at the individual level. Consequently, while BAG serves as a promising population-level metric for monitoring aging trajectories, these findings necessitate a cautious interpretation, acknowledging that statistical significance at the group level does not directly translate into diagnostic applicability for single subjects.

Despite these limitations, the observed BAG difference provides an additional cross-sectional neuroimaging metric that may complement conventional morphometric analyses in characterizing group-level neuroanatomical differences in adult ASD. Even a modest group-level shift suggests that the cumulative impact of neurodevelopmental atypicality may render the ASD brain more vulnerable to aging processes. Our findings suggest that the elevated estimated BAG in adults with ASD reflects a cross-sectional difference in global brain-age metrics, whereas the localized GMV and FD findings should be regarded as exploratory observations that require further replication. This is consistent with evidence from large-scale multicenter studies demonstrating widespread cortical morphological differences in ASD (Lesourd et al., 2023). Furthermore, the potential modifiability of this trajectory–as suggested by studies showing that aerobic exercise or cognitive training can mitigate BAG in other populations (Wan et al., 2026) –offers a compelling avenue for future research. Whether such neuroplasticity extends to adults with ASD remains unknown, warranting direct investigation through longitudinal or interventional studies to determine if these early structural features predict functional decline later in life.

Importantly, the harmonized BAG differences remained statistically significant after adjustment for chronological age, FIQ, and TIV. Furthermore, a TD-based residual bias-correction sensitivity analysis yielded comparable results, indicating that the observed group difference was not substantially altered after accounting for age-related prediction bias. Nevertheless, given the cross-sectional design and the inherent limitations of brain-age modeling, these findings should be interpreted as evidence of group differences in estimated brain-age metrics rather than definitive evidence of accelerated biological aging or specific neurobiological mechanisms.

### 4.3 Limitations

This study has several limitations. First, the absence of longitudinal data prevents us from characterizing developmental trajectories or temporal changes in brain structure among adults with ASD; consequently, our cross-sectional findings should be interpreted cautiously as a preliminary neurodevelopmental indicator rather than a definitive statement on the biological aging process itself. Second, although ComBat harmonization was applied to reduce site-related variability, the use of multi-site public data may still introduce residual site effects. Accordingly, the localized GMV and FD findings reported in the present study should be regarded as exploratory until replicated in independent cohorts with more balanced multi-site sampling. Sensitivity analyses performed using unharmonized data did not identify significant localized GMV or FD differences after multiple-comparison correction, suggesting that site-related variance substantially attenuates statistical power in high-dimensional neuroimaging analyses. These findings highlight the practical importance of harmonization procedures for multi-center datasets while also emphasizing that harmonization should be regarded as a strategy to mitigate, rather than eliminate, potential site-related confounding. Future studies employing larger and more diagnostically balanced multi-site samples will be important for further validating the present findings. Third, adult ASD samples are subject to various confounders. Although the dataset includes information on certain comorbidities (see Supplementary Material for details), other critical factors–such as medication history, therapeutic interventions, and employment status–were largely unavailable. The absence of these variables may confound the interpretation of our findings, necessitating more comprehensive phenotyping in future studies to better isolate core neurobiological effects. Fourth, brain age estimation was based on a pre-trained model, and thus model performance characteristics and bias-correction procedures relied on previously published validation rather than dataset-specific recalibration, which may introduce uncertainty when applied to independent cohorts. Fifth, our clinical characterization relied primarily on ADOS scores, which provides a limited assessment of ASD symptomatology. Finally, because only male participants were included, the findings may not generalize to females. Future research should recruit locally sampled and longitudinal cohorts, incorporate broader clinical assessments such as the Social Responsiveness Scale (SRS), the Autism Diagnostic Interview–Revised (ADI-R), and comprehensive cognitive or executive-function batteries, and ensure adequate representation of female participants to strengthen the stability, generalizability, and sex-related validity of structural markers in adult ASD.

## 5 Conclusion

By integrating brain age estimation with volumetric and surface-based morphometric analyses, our findings indicate cross-sectional differences in estimated BAG in adults with ASD relative to TD controls. This was accompanied by exploratory morphometric differences identified through volumetric and surface-based analyses. These findings provide a descriptive cross-sectional perspective on brain–behavior associations in this sample.

## Data Availability

Publicly available datasets were analyzed in this study. This data can be found here: Autism Brain Imaging Data Exchange (ABIDE).

## Footnotes

1
https://www.fil.ion.ucl.ac.uk/spm/

2
https://github.com/james-cole/brainageR

3
https://github.com/jfortin1/ComBatHarmonization/tree/master/R
