# Supplementary material for: Structural brain alterations associated with brain age may link to social dysfunction in male adults with autism spectrum disorder
Source: Front Neurosci. 2026 Jul 28;20:1795744. doi: 10.3389/fnins.2026.1795744 (PMC13457365; doi:10.3389/fnins.2026.1795744)
Supplement: Supplementary file 1 [file Data_Sheet_1.docx]

**Supplementary Table S1. Sample selection across ABIDE I sites**

| **Site** | **Original ABIDE** | **Excluded: Female** | **Excluded: Left-handed** | **Excluded: QC Failure** | **Excluded: Missing FIQ** | **Total Excluded** | **Final Included** | **Included: ASD** | **Included:**  **TD** | **Comorbidity** |
| --- | --- | --- | --- | --- | --- | --- | --- | --- | --- | --- |
| CMU | 27 | 6 | 0 | 0 | 0 | 6 | 21 | 10 | 11 | 0 |
| LEUVEN_1 | 29 | 6 | 3 | 1 | 6 | 16 | 13 | 0 | 13 | 0 |
| MAX_MUN | 42 | 7 | 3 | 5 | 6 | 21 | 21 | 0 | 21 | 0 |
| NYU | 49 | 2 | 2 | 2 | 9 | 15 | 34 | 11 | 23 | 4 |
| OLIN | 15 | 0 | 0 | 3 | 2 | 5 | 10 | 6 | 4 | 0 |
| PITT | 24 | 1 | 2 | 0 | 0 | 3 | 21 | 10 | 11 | 0 |
| SBL | 29 | 1 | 0 | 2 | 2 | 5 | 24 | 11 | 13 | 0 |
| TRINITY | 20 | 0 | 0 | 0 | 0 | 0 | 20 | 10 | 10 | 0 |
| USM | 66 | 2 | 2 | 2 | 2 | 8 | 58 | 32 | 26 | 0 |
| **Total** | 301 | 26 | 12 | 14 | 27 | 79 | 222 | 90 | 132 | 4 |

**Detailed information on the data acquisition sites is as follows: CMU (Carnegie Mellon University), LEUVEN_1 (University of Leuven: Sample 1), MAX_MUN (Ludwig-Maximilians-University Munich), NYU (NYU Langone Medical Center), OLIN (Olin, Institute of Living, at Hartford Hospital), PITT (University of Pittsburgh), SBL (Social Brain Lab), TRINITY (Trinity College Dublin), and USM (University of Utah School of Medicine). Among the participants included in the final analysis, four individuals had documented psychiatric comorbidities, which were: ADHD (inattentive type), dysthymia (two cases), and a concurrent diagnosis of dysthymia and agoraphobia.**

**Supplementary Table S2. Brain age prediction performance across diagnostic groups**

| Diagnostic Group | Sample Size (N) | Mean Absolute Error (Years) | Chronological vs. Predicted Age (r) | Mean Prediction Error（Years) |
| --- | --- | --- | --- | --- |
| TD Group | 132 | 3.51 | r = 0.789, p < 0.001 | +0.05 |
| ASD Group | 90 | 3.95 | r = 0.863, p < 0.001 | +1.48 |
| Overall | 222 | 3.68 | r = 0.808, p < 0.001 | +0.63 |

**Supplementary Table S3. Participant IDs for all subjects**

| Site ID | Included Subject IDs |
| --- | --- |
| CMU | 50642, 50643, 50645, 50646, 50647, 50649, 50651, 50652, 50653, 50655, 50657, 50658, 50659, 50660, 50663, 50664, 50665, 50666, 50667, 50668, 50388 |
| LEUVEN_1 | 50682, 50683, 50685, 50687, 50688, 50691, 50692, 50699, 50701, 50703, 50707, 50709, 50710 |
| MAX_MUN | 51332, 51333, 51334, 51335, 51338, 51339, 51340, 51341, 51342, 51343, 51344, 51345, 51346, 51347, 51362, 51364, 51365, 51367, 51369, 51371, 51373 |
| NYU | 51015, 51016, 51017, 51018, 51021, 51023, 51024, 51025, 51026, 51028, 51029, 51066, 51067, 51068, 51112, 51113, 51114, 51115, 51116, 51117, 51118, 51119, 51130, 51131, 51146, 51147, 51149, 51150, 51151, 51152, 51153, 51154, 51155, 51156 |
| OLIN | 50107, 50108, 50109, 20114,50121, 50124, 50130, 50134, 50136, 50137 |
| PITT | 50004, 50008, 50009, 50012, 50016, 50017, 50019, 50020, 50024, 50025, 50030, 50032, 50037, 50039, 50040, 50041, 50042, 50046, 50052, 50058, 50060 |
| SBL | 51556, 51557, 51558, 51559, 51560, 51561, 51562, 51563, 51564, 51565, 51566, 51567, 51568, 51572, 51576, 51576, 51576, 51576, 51577, 51578, |
|  | 51579, 51580, 51581, 51582 |
| TRINITY | 50232, 50233, 50236, 50237, 50238, 50244, 50245, 50246, 50253, 50254, 50259, 50260, 50261, 50262, 50263, 50264, 50270, 50271, 51132, 51139 |
| USM | 50434, 50439, 50440, 50442, 50444, 50445, 50446, 50449, 50450, 50452, 50454, 50455, 50457, 50458, 50459, 50462, 50463, 50465, 50466, 50467, 50468, 50469, 50471, 50472, 50473, 50474, 50475, 50476, 50477, 50478, 50479, 50480, 50482, 50483, 50484, 50485, 50487, 50488, 50490, 50491, 50492, 50494, 50496, 50497, 50498, 50499, 50502, 50503, 50505, 50507, 50508, 50514, 50521, 50525, 50526, 50527, 50529, 50531 |

**Supplementary Table S4. Sensitivity analysis of the regression model without harmonization**

| MLR | B | SE | β | t | p | 95% CI | VIF |
| --- | --- | --- | --- | --- | --- | --- | --- |
| Group (ASD vs. TD) | 1.849 | 0.753 | 0.169 | 2.456 | 0.015 | [0.365, 3.333] | 1.117 |
| Age | -0.059 | 0.051 | -0.077 | -1.153 | 0.250 | [-0.159, 0.042] | 1.053 |
| FIQ | 0.049 | 0.029 | 0.118 | 1.719 | 0.087 | [-0.07, 0.105] | 1.113 |
| TIV | -0.007 | 0.003 | -0.173 | -2.565 | 0.011 | [-0.013, -0.002] | 1.067 |
| SITE | 0.236 | 0.122 | 0.135 | 1.938 | 0.054 | [-0.04, 0.475] | 1.143 |

Note: For the "Group" variable, TD was coded as 1 and ASD was coded as 2.

**Supplementary Table S5. Sensitivity analysis of the regression model after exclusion of selected imaging sites**

| MLR | B | SE | β | t | p | 95% CI | VIF |
| --- | --- | --- | --- | --- | --- | --- | --- |
| Group (ASD vs. TD) | 1.890 | 0.747 | 0.184 | 2.532 | 0.012 | [0.417, 3.363] | 1.045 |
| Age | -0.066 | 0.051 | -0.092 | -1.291 | 0.198 | [-0.166, 0.035] | 1.003 |
| FIQ | 0.033 | 0.029 | 0.085 | 1.147 | 0.253 | [-0.024, 0.090] | 1.076 |
| TIV | -0.007 | 0.003 | -0.182 | -2.488 | 0.014 | [-0.013, -0.002] | 1.052 |

Note: For the "Group" variable, TD was coded as 1 and ASD was coded as 2.

### **Supplementary Table S6. Complete Statistical Specifications Matrix for Primary Analyses**

| Analysis Model | Dependent Variable (Y) | Primary Predictor (X) | Covariate Structure | Multiple Comparison Correction | Initial Threshold / Parameters |
| --- | --- | --- | --- | --- | --- |
| **Model 1:** Voxel-wise GMV Group Comparison | Local Voxel Gray Matter Volume maps | Diagnosis (ASD vs. TD) | Chronological Age, FIQ, TIV | **GRF** (Gaussian Random Field) | Voxel-level *P* < 0.001 (uncorrected); Cluster-level *p* < 0.05(corrected) |
| **Model 2:** Vertex-wise FD Group Comparison | Local Vertex Fractal Dimension maps | Diagnosis (ASD vs. TD) | Chronological Age, FIQ | **FDR** (False Discovery Rate) | Vertex-level *P* < 0.05 (FDR corrected); *No initial cluster threshold required* |
| **Model 3:** Global BAG Group Comparison | Estimated Brain Age Gap (scalar) | Diagnosis (ASD vs. TD) | Chronological Age, FIQ | *Not Applicable* (Single Scalar Metric) | Standard General Linear Model (GLM); Significance set at *p* < 0.05 |
| **Model 4:** Imaging–ADOS Association | Local Cluster GMV (Right Precuneus) | ADOS Social Affect Score | Chronological Age, FIQ, TIV | *Not Applicable* (Region-of-Interest Analysis) | Exploratory Partial Correlation; Significance set at *p* < 0.05 |
| **Model 5:** BAG–Morphology Association | Global BAG (scalar) | Local Cluster GMV / FD values | Chronological Age, FIQ | *Not Applicable* (Region-of-Interest Analysis) | Partial Correlation; Significance set at *p* < 0,05 |

**Supplementary Fig. S1. Normal Q-Q Plot of Regression Standardized Residuals. Comparing the observed cumulative distribution of the regression residuals against the expected normal distribution. The close alignment of the data points along the 45-degree diagonal line confirms that the residuals of the BAG model follow a normal distribution, satisfying the normality assumption for multiple linear regression.**

**Supplementary Fig. S2. Scatterplot of Standardized Residuals against Predicted Values. The relationship between the regression standardized residuals (Y-axis) and the regression standardized predicted values (X-axis) for the BAG model. The random and uniform distribution of residuals around the zero horizontal line, without any discernible funnel or curvilinear patterns, demonstrates homoscedasticity and confirms the linearity of the model**

**Supplementary Fig. S3. Group-stratified scatterplot of brain age against chronological age.**

**Supplementary Fig. S4. Site-stratified scatterplot of brain age against chronological age across imaging centers (with sample sizes greater than or equal to 20).**

**Supplementary Sensitivity and Diagnostic Analyses**

Brain age model performance was evaluated separately within the TD and ASD groups using the mean absolute error (MAE), mean prediction error (MPE), and the correlation between predicted and chronological age. The model demonstrated comparable performance across diagnostic groups (Supplementary Table 2). Predicted age showed strong correlations with chronological age in the overall sample (r = 0.825, p < 0.001), the TD group (r = 0.789, p < 0.001), and the ASD group (r = 0.863, p < 0.001).

A sensitivity analysis excluding Full-Scale Intelligence Quotient (FIQ) as a covariate was conducted to test the robustness of the primary findings. The group effect on BAG remained statistically significant (F = 5.356, p = 0.022, partial η² = 0.024), with higher BAG observed in the ASD group compared to TD controls (B = 1.546, SE = 0.668, 95% CI [0.229, 2.863]). The overall model was also significant (F = 3.247, p = 0.023, R² = 0.043). Multicollinearity diagnostics: tolerance range = 0.994–0.998, VIF range = 1.002–1.006. These results were consistent with the main analysis, indicating that the group effect was not dependent on the inclusion of FIQ as a covariate.

To assess potential age-related prediction bias, correlations between BAG and chronological age were examined in the overall sample and within each diagnostic group. No significant association was observed in the overall sample (r = -0.037, p = 0.585), the TD group (r = -0.121, p = 0.169), or the ASD group (r = -0.063, p = 0.562), indicating minimal residual age dependency in BAG estimates.

Site-stratified evaluations of brain age model performance were conducted across major imaging sites, and parallel analyses without ComBat harmonization were additionally performed. Site-wise correlations between predicted brain age and chronological age are presented in Supplementary Figure S4. Collectively, these supplementary results suggest that the brain age model demonstrates stable performance across diagnostic groups and imaging sites, reducing the likelihood that the observed BAG differences are driven by site-related or model-related artifacts.

Parallel analyses performed without ComBat harmonization yielded consistent findings with the primary results. The diagnostic group effect remained robust and statistically significant (F = 6.032, p = 0.015, eta^2^ = 0.027), supporting the stability of the observed group differences.

The primary regression analysis was repeated after excluding participants from the LEUVEN_1 and MAX_MUN sites. The diagnostic group remained a significant predictor of BAG after adjustment for chronological age, TIV, and FIQ (Supplementary Table S5). The overall regression model remained statistically significant (F = 6.511, *p* = 0.014, eta^2^ = 0.0340), and the direction and magnitude of the group effect were comparable to those observed in the primary analysis, supporting the stability of the main findings.

Model assumptions were evaluated through a series of diagnostic procedures. Quantile–quantile (Q–Q) plots demonstrated that the residuals closely followed the theoretical normal distribution. Homoscedasticity was formally assessed using the Breusch–Pagan test, which did not indicate significant heteroscedasticity (χ² (1) = 0.073, p = 0.786). Potentially influential observations were evaluated using centered leverage statistics, and no multivariate outliers exceeded acceptable thresholds (maximum centered leverage = 0.147).

In addition, a TD-based residual bias-correction analysis was performed. A linear regression model predicting BAG from chronological age was estimated in the TD group and subsequently applied to the entire sample. Residual BAG values were calculated as the difference between observed and age-predicted BAG. The ASD group continued to show significantly higher residual BAG values than the TD group (t = -2.230, p = 0.027, Cohen's d = -0.305, CI [0.035,0.574]).

To examine the influence of extreme observations, the primary analyses were repeated after excluding participants with BAG values exceeding ±2.5 standard deviations from the sample mean. brain age effect in the ASD cohort remained stable and consistent (F = 3.968, p = 0.048, eta^2^ = 0.018). The direction and statistical significance of the main findings remained unchanged, indicating that the results were not driven by a small number of extreme observations.

**Supplementary brainageR detailed description**

Software for generating a brain-predicted age value from a raw T1-weighted MRI scan. This uses a Gaussian Processes regression, implemented in R, using the kernlab package.

The software takes raw T1-weighted MRI scans, then uses SPM12 for segmentation and normalisation. A slightly customised version FSL *slicesdir* is then used to generate a directory of PNGs and corresponding index.html file for quality controlling in a web browser. Finally, the normalised images and loaded into R using the RNfiti package, vectorised and grey matter, white matter and CSF vectors masked (using 0.3 in the average image from the brainageR-specific template, derived from n=200 scans, n=20 from each of the n=10 scanners) and combined. In version 2.0 Principal Components Analysis was run (using R's prcomp), and the top 80% of variance retained. This meant 435 PCs were included. The rotation matrix of the PCA is applied to any new data adn these 435 variables are then used to predict an age value with the trained model with kernlab. The entailed using a GPR with RBF kernel and default hyperparameters.

The brainageR model for v2.1 was trained on n = 3377 healthy individuals (mean age = 40.6 years, SD = 21.4, age range 18-92 years) from seven publicly-available datasets, and tested on n = 857 (mean age = 40.1 years, SD = 21.8, age range 18-90 years). All participants included were healthy according to local study data. For example, OASIS participants were only included if they had a CDR score < 0.5. All data was visually quality control to ensure quality and accuracy of image processing. Demographics were error-checked, and exclusions made if age values were unavailable.

The model performance on the held-out test data (with random assignment to training and test) is as follows: Pearson's correlation between chronological age and brain-predicted age: r = 0.973, mean absolute error = 3.933 years, R^2^ = 0.946. While a bias has been reported in terms of a correlation between chronological age and the brain-age difference, in this GPR model the correlation in the test set was r = -0.012. Hence, the model DOES NOT automatically correct predictions for a statistical dependency on chronological age. It is still recommend to use age as a covariate in future analysis that use brain-prediced age difference (brain-PAD) as the outcome measure.

The above information is clearly described in the official brainageR documentation and user guidelines:

(https://github.com/james-cole/brainageR).

**Supplementary: Algorithmic Implementations and Analytical Scripts**

To ensure computational transparency, this section details the operational syntax for brain-age prediction, multi-site ComBat harmonization, and the statistical setups mapped across the R, MATLAB (CAT12), and SPSS platforms.

#### **1. Brain Age Prediction via brainageR (R Environment)**

Brain age estimation was performed using **brainageR version 2.1** (<https://github.com/james-cole/brainageR>), a publicly available framework for estimating brain-predicted age from structural MRI data.

The software environment consisted of:

- MATLAB (2023a)
- SPM12 (revision r7771)
- R (version 4.3.2)
- R packages: kernlab, RNifti, stringr
- FSL (used for quality-control image generation)

The pretrained brainageR v2.1 model was used directly without retraining, parameter optimization, or transfer learning.

Chronological aging structural footprints were translated into estimated brain age using the pre-trained Gaussian Process Regression framework implemented in the brainageR package.

```
library(brainageR)
% Preprocessed and segmented structural T1 NIfTI volumes were mapped to the brainageR pipeline
brain_age_results <- brainage(
  x = "/path/to/segmented_T1_images.nii",
  outdir = "/path/to/output_directory/"
)
% Read merged prediction table
brain_age <- read.csv("brain_predicted_age.csv")
% Extracting raw estimated brain age vector
estimated_brain_age <- brain_age_results$brain_age
```

#### **2. Multi-Site ComBat Harmonization (R Implementation)**

Both high-dimensional imaging maps and the univariate scalar BAG metrics were harmonized via the native R implementation of ComBat to mitigate cross-site scanner configurations.

*% Obtaining the native script from (*[*https://github.com/jfortin1/ComBatHarmonization/tree/master/R*](https://github.com/jfortin1/ComBatHarmonization/tree/master/R)*)*

*source("combat.R")
% Matrix setup: 'dat' represents the neuroimaging feature matrix (voxels/vertices or scalar BAG vector)
% 'batch' represents the multi-site categorical vector
% 'mod' designates the biological model matrix protecting clinical diagnosis
mod <- model.matrix(~Diagnosis + Chronological_Age + FIQ, data = sample_metadata)
batch <- sample_metadata$Scan_Site
% Executing empirical Bayes distribution alignment
harmonized_data <- comBat(dat = neuroimaging_matrix, batch = batch, mod = mod)*

#### **3. SPSS Statistical Analysis Workflow**

Software Environment

Statistical analyses were performed using IBM SPSS Statistics Version 29.0 (IBM Corp., Armonk, NY, USA).

Unless otherwise specified, all statistical tests were two-tailed and statistical significance was defined as p < 0.05.

Demographic and Clinical Comparisons

Continuous variables were compared between ASD and TD groups using independent-samples t-tests.

Variables included:

Age

Full-scale IQ (FIQ)

Total intracranial volume (TIV)

Brain-age gap (BAG)

Example SPSS syntax:

T-TEST GROUPS

Categorical variables were compared using χ² tests.

Primary BAG Group Comparison

To examine group differences in BAG, multiple linear regression was performed with BAG as the dependent variable and diagnostic group as the primary predictor.

Model:

BAG = β0 + β1 (Group) + β2 (Age) + β3 (TIV) + β4 (FIQ) + ε

Example SPSS syntax:

REGRESSION

/MISSING LISTWISE

/STATISTICS COEFF OUTS R ANOVA

/CRITERIA=PIN (.05) POUT (.10) TOLERANCE (.0001)

/NOORIGIN

/DEPENDENT BAG

/METHOD=ENTER group age tiv FIQ.

Sensitivity Analysis Without FIQ

To evaluate the influence of intellectual functioning on the primary BAG findings, the regression model was repeated after removing FIQ from the covariate structure.

Model:

BAG = β0 + β1(Group) + β2(Age) + β3(TIV) + ε

Example SPSS syntax:

REGRESSION

/DEPENDENT BAG

/METHOD=ENTER group age tiv.

Outlier-Exclusion Sensitivity Analysis

Sensitivity analyses were repeated after excluding predefined outlier observations.

The same regression framework was applied to the reduced dataset.

Model specification remained identical to the primary BAG model.

TD-Based Residual BAG Sensitivity Analysis

To evaluate the potential influence of age-related prediction bias, a linear regression model was first estimated within the TD group:

BAG = β0 + β1(Age) + ε

Predicted BAG values were generated from this model.

Residual BAG values were then calculated as:

Residual BAG = Observed BAG − Predicted BAG

Residual BAG values were subsequently compared between ASD and TD groups.

Example SPSS procedure:

Analyze

→ Regression

→ Linear→ Save

→ Unstandardized Predicted Values

Then:

Transform

→ Compute Variable

Residual_BAG = BAG − PRE_1

Correlation Analysis

Pearson correlation analyses were performed to evaluate associations between:

BAG and chronological age

Residual BAG and chronological age

Example SPSS syntax:

CORRELATIONS

/VARIABLES=age BAG residual_BAG

/PRINT=TWOTAIL NOSIG.

Partial Correlation Analysis

Associations between imaging metrics and clinical measures were evaluated using partial Pearson correlations.

Analyses included:

GMV and ADOS scores

FD and ADOS scores

BAG and GMV measures

BAG and FD measures

Covariates:

Age

FIQ

TIV

Example SPSS syntax:

PARTIAL CORR

/VARIABLES=GMV ADOS

/CONTROLLING=age FIQ TIV.

Effect Size Estimation

Group differences were additionally quantified using standardized effect sizes.

Effect sizes included:

Cohen’s d

Hedges’ g

Glass’s Δ

These were generated using:

Analyze

→ Compare Means

→ Independent-Samples T Test

→ Options

→ Effect Size

Graphical Visualization

Scatter plots were generated for:

Brain age versus chronological age

BAG versus chronological age

Residual BAG versus chronological age

Example SPSS procedure:

Graphs

→ Chart Builder

→ Scatter/Dot

→ Simple Scatter

Linear fit lines and 95% confidence intervals were added where appropriate.

#### **4. Voxel-wise and Vertex-wise General Linear Models (MATLAB / CAT12 Engine)**

For high-dimensional brain maps (Model 1 and Model 2), general linear modeling is structured via the statistical module of the CAT12/SPM12 engine in MATLAB prior to multi-model pooling under the FDR family.

```
% Parametric General Linear Model layout within CAT12 Batch Architecture
matlabbatch{1}.spm.stats.factorial_design.dir = {'/path/to/CAT12_stats/'};
% Defining the non-independent factor cells (ASD group vs. TD group scans)
matlabbatch{1}.spm.stats.factorial_design.des.t2.scans1 = {'/path/to/ASD_harmonized_maps.nii'};
matlabbatch{1}.spm.stats.factorial_design.des.t2.scans2 = {'/path/to/TD_harmonized_maps.nii'};
% Covariates configuration Matrix
matlabbatch{1}.spm.stats.factorial_design.cov(1).c   = [Age_Vector];
matlabbatch{1}.spm.stats.factorial_design.cov(1).cname = 'Age';
matlabbatch{1}.spm.stats.factorial_design.cov(2).c   = [FIQ_Vector];
matlabbatch{1}.spm.stats.factorial_design.cov(2).cname = 'FIQ';
% Total Intracranial Volume (TIV) vector covariate - SPECIFICALLY INCLUDED ONLY FOR VOXEL-WISE GMV (MODEL 1)
matlabbatch{1}.spm.stats.factorial_design.cov(3).c   = [TIV_Vector];
matlabbatch{1}.spm.stats.factorial_design.cov(3).cname = 'TIV';
spm_jobman('run', matlabbatch);
```
